# Utilization of Recycled Liquid Crystal Display (LCD) Panel Waste in Concrete

**DOI:** 10.3390/ma12182941

**Published:** 2019-09-11

**Authors:** Jacek Góra, Małgorzata Franus, Danuta Barnat-Hunek, Wojciech Franus

**Affiliations:** 1 Department of Construction, Faculty of Civil Engineering and Architecture, Lublin University of Technology, Nadbystrzycka 40, 20-618 Lublin, Poland; j.gora@pollub.pl (J.G.); m.franus@pollub.pl (M.F.); d.barnat-hunek@pollub.pl (D.B.-H.); 2 Department of Geotechnical Engineering, Faculty of Civil Engineering and Architecture, Lublin University of Technology, Nadbystrzycka 40, 20-618 Lublin, Poland

**Keywords:** waste liquid crystal displays, recycling, concrete, micro-structure, frost resistance, water-tightness

## Abstract

The paper presents the possibility of using the liquid crystal display (LCD) waste as a partial substitute of fine aggregate. Concretes with two types of cement, CEM I 42.5 R and CEM II/B-S 42.5 N, with and without LCD addition, were investigated. The properties that influence the structures exposed to severe environments were examined. The results and analyses pertaining to their micro-structure, including interfacial transition zone (ITZ), were presented as well. All concretes exhibited good freeze–thaw (F–T) resistance after 150 F–T cycles. The water-tightness was established as 0.8 MPa. All concretes both with and without LCD achieved the same class C50/60.

## 1. Introduction

Technological development and improving standards of life contribute to an increasingly frequent use of liquid crystal and plasma display television sets, cell phones, tablets and personal computers [1,2]. It is estimated that the lifespan of these devices amounts to 3–8 years [3]; therefore, the amount of wastes from these devices is on a constant increase, of which the display panel constitutes over 40% [4,5]. In Europe, approximately 25.000 m^3^ of waste from PCs and TVs is produced per million people each year [1].

Glass screens constitute one type of the wastes originating from liquid crystal display (LCD) panels and television sets; hence, waste glass recycling at an industrial scale seems to be a very advantageous solution [6,7], including the civil engineering sector (construction aggregate recycling), secondary product manufacturing and production of other feedstocks [8,9]. The literature reports the recycling of car glass [10], soda-lime glass from containers [11,12,13] glass from the process lines of the pharmaceutical industry [14,15,16] and cathode ray tubes of television sets and computer monitors [17,18]. The issue of storage can be solved by grinding glass to powder fraction and adding it to concrete as a pozzolanic material [19].

The conducted literature study indicated numerous papers on the possibility of applying broken glass in concrete and related products. Bhandar and Tajne [20], Degirmenci et al. [21] and Terro [22] mention that using monitor waste as an aggregate can reduce the mechanical strength of composites when sand is substituted with glass in the amount greater than 10–30%. Studies indicate that comparable or greater tensile and flexural strengths may be achieved if the samples contain 20–25% monitor wastes. When wastes are used as cement substitute in concrete, the proposed optimal amount varies and ranges from 10% [23] to 20% [24] or even up to 40% [25]. Ling and Poon [26] as well as Terro [22] report that owing to the smooth and impermeable surface of fine glass pebbles, using this type of waste as an aggregate may improve the liquidity of fresh mortar. The application of waste glass as aggregate may reduce the high water requirement of nanosilica. Therefore, *n*SiO_2_ may be successfully added to cement composites without additional dispersing agents [27]. Aliabdo et al. [28] state that in the concretes in which cement was substituted with broken glass in the amount of 10%, following 28 days of maturation, the compressive strength of 30 MPa and tensile strength of 4 MPa were obtained, while density (2290 kg/m^3^) increased by about 9.0%. When glass powder in the amount of 15% was used as cement additive, the compressive strength improved to 38 MPa and tensile strength increased to 3.8 MPa; in turn, absorption and tightness of concrete decreased by 16.0%, on average. The studies conducted by Corinaldesi et al. [29] indicate that the compressive strength of concrete after 28 days of maturation, in which the quartz sand was substituted with colorless broken glass in the amount of 50% reaches about 30 MPa, while the tensile strength equals 5.9 MPa and is comparable to the reference concrete. In the brief induction of glass powder in concrete, Du and Tan [30,31] have attempted to replace cement by glass powder by 60% in concrete. Good mechanical properties and durability have been reported by those studies.

LCDs have a complex composition because a device typically consists of the following sections: the front panel, the LCD panel, the plastic housing, a series of films, the back panel, the backlight origin, the power supply and control, a rear cover and a base. The components of the LCD panel include glass (85–87%), polymer membrane (12.7–14%), and liquid crystals (0.12–0.14%) [32]. A liquid crystal is composed of glass substrates, liquid crystal, ITO conductive glass and black matrix (chromium oxide), and is characterized as an interim state between solid and liquid.

In 2018, Amato and Beolchini [33] presented a comprehensive literature review involving the international patents about LCD recycling from 1999 to 2017. The inventions included waste disassembling, followed by the classification of the target fractions and the recovery of metal (e.g., indium, indium tin oxide) and nonmetal (e.g., glass, polarizing film, liquid crystal) components.

Thorough literature studies showed that different wastes were used as an aggregate substitute in concrete production [34,35], but there are no studies concerning the use of liquid crystal display panel parts for this purpose. The paper presents the possibility of using LCD panel parts in concrete production. Additionally, the influence on physical, mechanical and structural properties was analyzed.

## 2. Experimental Materials

### 2.1. Aim and Scope of the Experiment

The goal of this study involved investigating and assessing the characteristics of concretes with the ground LCD waste addition. In order to conduct a comparison, identical experiments were performed for the non-modified concretes. Concretes with two types of cement, i.e., CEM I 42.5 R and CEM II/B-S 42.5 N, were investigated to broaden the scope of research. Apart from the mechanical properties of concretes, the properties corresponding to durability of products and structures under harsh climatic conditions were examined as well. In order to explain the differences in concrete behavior, the paper additionally presented the results of studies and analyses on their micro-structure.

### 2.2. Materials

The chemical composition of cement clinker is presented in Table 1 and Table 2. In turn, the physical properties of cement are shown in Table 3 and Table 4.

The fly ash used in the research came from the combustion of coal at the Kozienice Power Plant (Świerże Górne, Poland). The chemical composition of this fly ash consists predominately of SiO_2_ (52.12%), Al_2_O_3_ (32.19%) and Fe_2_O_3_ (5.17%). The sum of main oxides indicates that the tested fly ash is an F-class fly ash (alumino-silicated) according to ASTM C618-08 [36]. Race components included: MgO (1.29%), CaO (1.16%), Na_2_O (0.49%), K_2_O (2.87%), TiO_2_ (1.38%), P_2_O_5_ (0.43%), LOI = 2.60%. Weight percentages of other components were determined following the EN 450 [37], EN 451 [38] standards: chlorides (0.001–0.01 wt.%), free CaO (0.02–0.10 wt.%), pH 10.7. The mineral composition was dominated by spherical forms of aluminosilicate glass (69.8%) and mullite (21.2%). In addition, quartz (7.4%) and iron oxides (1.6%) were observed on the surface of the aluminosilicate spheres. Fly ash was employed because the concretes with this additive (in the amount up to 20%) are characterized by higher strength and resistance to cracking, corrosion and high temperatures [39]. Natural rinsed quartz sand and natural gravel were utilized for concrete preparation. The density of these aggregates amounted to 2.65 kg/dm^3^. The particle size gradation of the fine and coarse aggregate, as well as LCD waste, are shown in Table 5. The density of LCD waste amounted to 2.49 kg/dm^3^.

In total, eight concrete mixtures were prepared; their composition is shown in Table 6. The mixtures were prepared using CEM I 42.5 R (marked as CI) and CEM II/B-S 42.5 N (marked as CII); two of them lacked the ground LCD admixture (CI.0 and CII.0), while in the remaining ones, the addition amounted to 1% (CI.1 and CII.1), 2% (CI.2 and CII.2) and 3% (CI.3 and CII.3) of cement mass. All the concrete mixtures had the same w/c ratio, equal to 0.34. Spent, ground LCD panels were used by substituting part of fine aggregate in concrete mixtures.

The following chemical admixtures were applied: polycarboxylate superplasticizer, chloride-free plasticizer, aerating admixture based on non-ionic surface active agents and liquid nanopolymer hydrophobizing agent based on an aqueous silane solution.

## 3. Research Method

### 3.1. X-ray Fluorescence (XRF)

The chemical composition of an LCD panel was determined with the XRF method, using a Philips PW 1404 spectrometer (Amsterdam, The Netherlands). A Cr–Au dual anode tube with the maximum constituted the maximum output of 3 kW constituted the excitation source.

### 3.2. X-ray Diffraction (XRD)

The mineral composition of waste materials, i.e., spent and ground LCD panels was analyzed with X-ray powder diffraction (XRD) method using the X’pert PROMPD spectrometer manufactured by Panalytical (Almelo, The Netherlands). The spectrometer was equipped with a PW 3050/60 goniometer (Panalytical), a graphite monochromator (Almelo, The Netherlands) as well as a Cu lamp. The analysis was conducted in the 5°–65° angle range (2θ). The obtained diffraction data was processed using Philips X’Pert Highscore software (High Score Plus v. 4.1). The mineral phases were identified on the basis of the PDF-2 database (release 2010), formalized by the ICDD.

### 3.3. Properties of Fresh Concrete

The consistency of concrete mixtures was tested in accordance with EN 12350-3 standard [40]. The content of air in the concrete mixtures was investigated in accordance with the EN 12350-7 standard [41]. The density of concrete mixtures was examined according to the PN-EN 12350-6 standard [42].

### 3.4. Properties of Hardened Concretes

The physical properties were determined in the following way: three cubic samples (edge length—150 mm) were produced from each concrete in order to conduct water absorption by weight as well as the volumetric density tests. Moreover, three cubic samples (edge length—150 mm) were prepared to conduct the porosity tests, in addition to six cubic samples (edge length—150 mm) which were used to test the water tightness and twelve cubic samples (edge length—100 mm) employed to investigate the frost resistance. The mechanical properties of each concrete were determined using eighteen cubic samples (edge length—100 mm) for conducting the compressive strength tests (six samples for tests following each period of 7, 28 and 90 days of maturation), six cuboid samples with the dimensions of 100 × 100 × 500 mm^3^ for the flexural strength test and six cubic samples with the edge length of 150 mm for splitting tensile strength test. Prior to testing, all samples maturated in water at the temperature of 20 ± 2 °C.

The samples for the water absorption test were suspended on a 10 mm mesh above the bathtub bottom and then poured with water up to half of their height. After 24 h, the water level was increased until it was 10 mm above the samples. After each subsequent 24 h, the samples were removed from water, their surfaces dried and then weighed. The concrete samples were saturated until the two consecutive weighings showed no mass increase. Following saturation, drying to constant mass and weighing of the samples were conducted, then the volumetric density and water absorption by weight which characterized the concrete were indicated in line with the PN-B-06250 standard [43].

When the samples were placed into the device for water-tightness testing, the pressure of water acting on the samples was increased gradually, every 24 h by 0.2 MPa, until 0.8 MPa was reached, which was maintained for the next 24 h. Throughout the experiment it was observed whether or not the water seeped through the samples. After the experiment, the samples were split and the depth of water infiltration into the concrete was determined. The adopted procedure was in accordance to the PN-B-06250 standard [43].

The frost resistance test was conducted after saturating the samples with water, similarly to the water absorption test. Freezing was carried out for 4 h at the temperature of −20 °C; then, the samples were thawed in water heated to +20 °C. Evaluation focused on the mass loss of the samples as well as the drop in the compressive strength in comparison to the control samples which were stored in water, at +20 °C ± 2 °C throughout the study. The procedure was carried out in compliance with PN-B-06250 [43].

The compressive strength test was performed according to EN 12390-3 [44] on cubic samples with the edge length of 100 mm. In order to determine the concrete classes, the results were calculated for the cubic samples with the edge length of 150 mm. The relationship characterizing the values of compressive strength in particular samples was adopted in accordance with PN-B-06250 [43] *f*_c,cube#150_ = 0.9 × *f*_c,cube#100_, where: *f*_c,cube#150_, *f*_c,cube#100_ are the compressive strengths observed on cubic samples with edge lengths of 150 and 100 mm, respectively. The tensile strength in bending was tested according to the procedure described in EN 12390-5 [45], while splitting tensile strength was investigated in accordance with EN 12390-6 [46].

### 3.5. Scanning Electron Microscopy with Energy Dispersive Spectroscopy (SEM/EDS)

The morphology of lightweight aggregates and their porous structure was determined using scanning electron microscopy (SEM). The observation was carried out by means of FEG Quanta 250 microscope (FEI, Hilsboro, OR, USA) equipped with an energy dispersive spectroscopy (EDS)-based system by EDAX for chemical composition analysis. The powder samples for SEM investigations were glued to a carbon holder using carbon glue. Then, the preparations were sputter coated with an approximately 50 nm layer of carbon in order to achieve conductivity on the sample surface.

### 3.6. Leaching Tests

Various methods for assessing the level of heavy metal leaching from building materials to the environment are employed in practice. Due to the lack of standardization of such methods [47,48], aqueous extract from concretes were prepared to determine the amount of leached heavy metal, in accordance with PN-EN 12457-4:2006 extract [49]. Redistilled water was added to the granulate in order to obtain the liquid/solid (*L*/*S*) phase ratio *L*/*S* = 100 mL⋅10 g of Solid 1. The samples were placed in 250 mL bottles and shaken in a rotating shaker for 24 ± 0.5 h at the speed of 200 r.p.m.). The trials were performed in triplicate at the temperature of 20 °C. The obtained liquid was filtered through a 0.45 μm membrane filter and then the aqueous extract was analyzed in terms of the heavy metal content using an ICP-OES spectrometer (Jobin-Ywon U-238, Longjumeau, France).

The second method for evaluating the leaching of heavy metals is the method maximum of leaching (ML) [50,51]. This method determines the possibility of maximum toxin leaching from the LCD panel under extreme environmental conditions [52]. The procedure was as follows: 500 mL of distilled water acidified to pH = 4 using HNO_3_ was added to 5 g of the sample ground to the fraction smaller than 125 µm. The sample prepared in this way was shaken for 5 h in the rotating shaker, while the solution pH was maintained throughout using 1 N HNO_3_. The concentrations of elements in eluates were determined by means of ICP-OES spectrometer (Jobin-Ywon U-238).

## 4. Results and Discussion

### 4.1. Chemical and Mineralogical Properties

The chemical analysis of an LCD panel indicated that silicon dioxide, SiO_2_, is the dominant component, constituting 24.64% of the weight (Table 7). The second most prominent component is aluminum oxide, which corresponds to 5.10% (*w*/*w*); calcium oxide constituted about half of this value, i.e., 2.68% (*w*/*w*). Arsenic, zinc, strontium, barium, phosphorus, potassium, iron, titanium, copper and indium oxides were found in trace amounts. The presence of hydrocarbons (organic carbon bonds) was found in LCD panels, reaching 65.12% (*w*/*w*).

**Figure 1:**
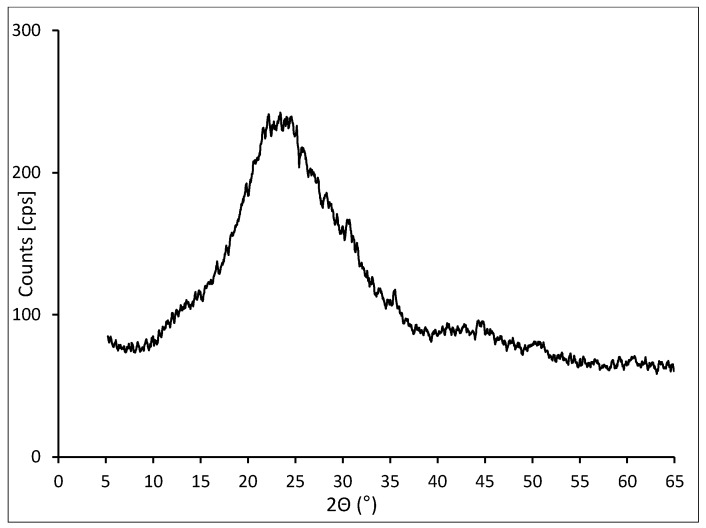
X-ray diffraction pattern of LCD material.

The mineral composition of the waste was analyzed with the powder method on the fraction of material smaller than 75 μm. The analysis indicated that amorphous silica SiO_2_ constitutes the dominant component, which was identified by a significant background increase at 2θ 15°–35° (Figure 1). The organic carbon bonds that did not form crystalline mineral phases remained unidentified in an investigation conducted with the XRD method.

**Figure 2:**
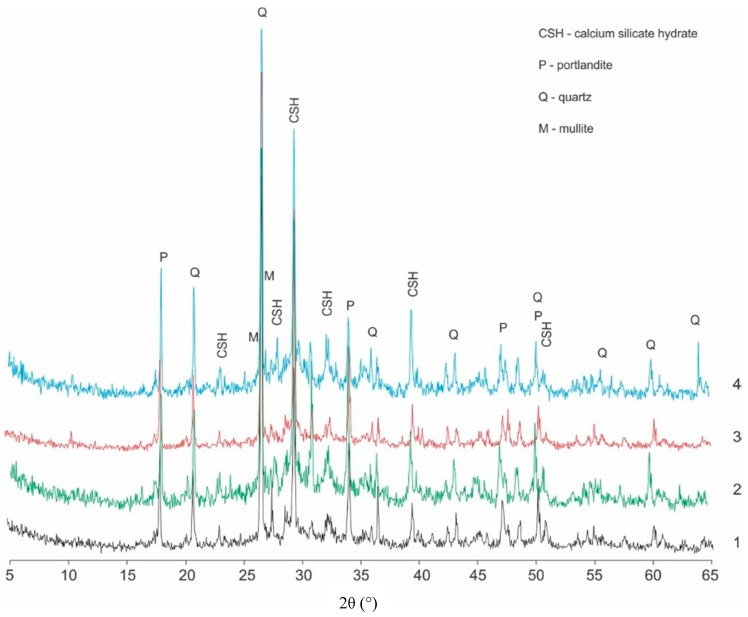
X-ray diffractograms of cement paste of (1) CI.0; (2) CI.1; (3) CII.0; and (4) CII.1 concretes.

The analysis of diffractograms confirms that in all concrete samples, the main hydration product involves phases of hydrated calcium silicates (C–S–H phase) in varying degrees of ordering. The results of reference analyses with 1% LCD addition are presented in Figure 2. Background intensities are especially high in the case of CI.1 and CII.1 concretes with LCD addition which can be observed at 2θ approximately 20°–35°. It can be a result both by the crystallization of poorly ordered C–S–H phases as well as by the addition of amorphous LCD (Figure 1). Additionally, intensities of peaks related to C–S–H phases in relation to peaks which can be observed on diffractograms of concretes without LCD panel addition (CI.0 and CII.0) are also higher. The main reason is probably the hydration reaction between glassy part of LCD panels and cements.

Peaks from crystalline intrusions, i.e., portlandite and quartz, were also identified in the considered cement paste samples. Crystalline phases were identified by the highest interplanar spacings *d_hkl_*, which reached a *d_hkl_* of 4.9 Å for portlandite and *d_hkl_* values of1.817, 3.346 and 4.26 Å for quartz. The presence of calcium carbonate in the form of calcite was found in the pastes separated from concrete, as a product of partial carbonatization. It was identified by the basic interplanar spacings *d_hkl_*, which amounted to 2.095, 2.285 and 3.035 Å (Figure 2).

### 4.2. Physical Properties

The results of studies conducted on fresh concrete mixtures are presented in Table 8. The obtained mean results pertaining to the physical properties of concretes are shown in Table 9.

The air content in concrete mixtures obtained using aerating admixture exceeds 4.0% (Table 8), which is currently required in the exposure classes from XF2 to XF4 (aggression caused by freezing/thawing with the presence of deicing agents or lack thereof), in accordance with EN 206 [53].

The results of studies on porosity indicate an advantageous distribution, size and content of pores in all considered concretes. Total air content *A* in concretes exceeds 5% (In Danish requirements, it should amount at least to 3.5%, regardless of the exposure class [55]), pore spacing factor *L* is lower than 0.2 mm (according to Danish [55] and German requirements, this is the maximum permissible value [56]), and the *A*_300_ (the percentage content of micropores with the diameter up to 300 μm) micropore content exceeds 2% (the Austrian [57] and German requirements provide the minimum value of 1.8% [56]).

Water absorption by weight obtained in the course of studies is lower than 6%. In line with the standards related to precast concrete paving elements (cobblestone, concrete curbs [57,58,59,60]), the following requirements must be met in terms of water absorption: Class 1, construction mark A—not determined; Class 2, construction mark B—mean value ≤ 6%. Because none of the obtained results exceed 5%, they should be considered satisfactory in relation to any class.

**Figure 3:**
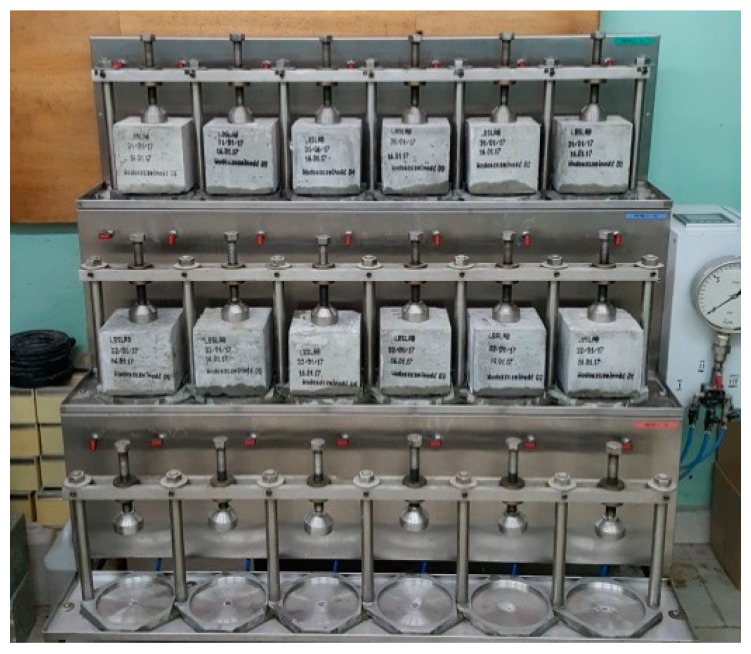
Water-tightness test of concretes.

In the water permeability tests (Figure 3) the samples were acted upon with the pressure up to 0.8 MPa, which corresponds to W8 water tightness class, according to PN-B-06250 standard [43]. No seepage of water through the samples under the maximum set pressure was observed; hence, the water-tightness class was determined as W8. Following the experiment, the samples were split and the depth of water infiltration into the concrete structure was determined. The depth of infiltration does not exceed 1.5 cm in any sample, which should be considered a very good and satisfactory result. It should be emphasized that the limit value of water absorption by weight amounting to 5% and minimum W6 water-tightness class are required in the case of concretes intended for bridge construction [61], which are considered to be exposed to the harshest environmental conditions and heaviest wear.

**Figure 4:**
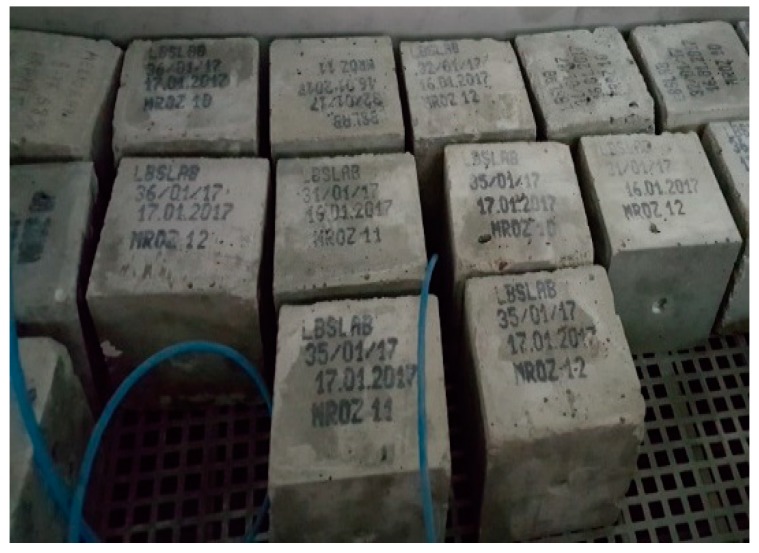
Frost resistance test of concretes.

The results obtained from the frost resistance test were evaluated in accordance with PN-B-06250 [43] (Figure 4), which involved examining the samples and checking their mass every 25 cycles. The compressive strength tests of frozen and unfrozen samples were conducted after 150 cycles, which corresponds to the highest degree of ordinary concrete frost resistance, in line with PN-B-06250 standard [43]. The considered samples showed no cracks, mass loss greater than 5% (the obtained values were insignificant, <0.5%); compressive strength reduction greater than 20%. High freezing–thawing resistance depends on low permeability and a low water/binder ratio. The resistance of concrete after 150 F–T cycles decreased by up to 3.8% in the case of CI and 0.7% for CII with CEM II/B-S 42.5 N. On the basis of the study results and above-mentioned criteria, the frost-resistance of considered concretes was determined as F150. Small-sized concrete elements are often exposed to aggressive impacts from the environment and thus they must have high resistance to frost corrosion, weathering or the influence of aggressive water. The studies confirmed a very high frost resistance and protection against water infiltration.

### 4.3. Strength Properties

**Figure 5:**
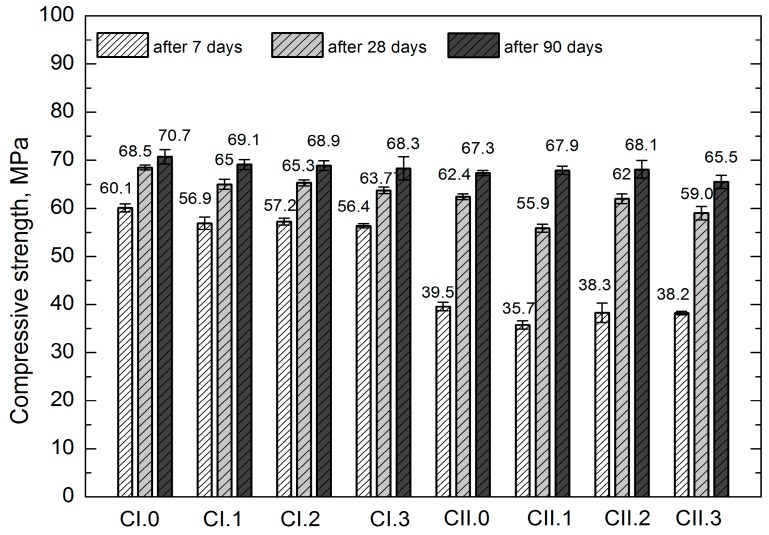
Compressive strength of concrete, *f*_c,cube #150_.

Strength tests were conducted following 7, 28 and 90 days from the sample molding. The mean values of mechanical properties of concretes are presented in Table 10 and Figure 5.

Prior to analyzing the results it should be emphasized that the requirements pertaining to the mechanical properties stated in the standards for precast concrete paving elements (cobblestone, concrete curbs) are related to the ready-made products. Due to their sizes that significantly differ from those of the standard samples employed in our investigations, conducting an analysis involving direct comparison of the obtained results and the standard requirements of particular products is impossible.

On the basis of the obtained results it was observed that all concretes after 28 days of maturation are characterized by the compressive strength *f*_c,cube#150_ greater than 55 MPa, and *f*_c,cube#100_ higher than 60 MPa. In light of the standards related to precast concrete paving elements (cobblestone, concrete curbs), the compressive strength is not accounted for in requirements and standard recommendations. It is only possible to refer to the previous versions of German standards, where—in the case of cobblestone—the compressive strength of the product should be higher than 60 MPa, and individual results should be greater than 50 MPa, in accordance with DIN 18501 [62]. Therefore, the *f*_c,cube#100_ results (more applicable, because the height of cobblestones usually does not exceed 100 mm) obtained from all studied concretes should be considered satisfactory. It should be noted that following 90 days of maturation, all concretes were classified as C50/60, i.e., higher than or equal to the class obtained after 28 days.

The requirements related to tensile strength in bending of cobblestone and concrete curbs are as follows: Class 1, construction mark S—characteristic strength 3.5 MPa, and minimal 2.8 MPa; Class 2, construction mark T—characteristic strength 4.0 MPa, and minimal 3.2 MPa, Class 3, construction mark U—characteristic strength 5.0 MPa, and minimal 4.0 MPa [59,60]. Since all the obtained results exceed 7 MPa, they should be considered satisfactory in relation to all classes of these elements.

The requirements for splitting tensile strength of cobblestone are as follows: characteristic strength of at least 3.6 MPa, individual results cannot be lower than 2.9 MPa [58]. The results obtained in the research exceeded 4 MPa and thus they are satisfactory. Similar findings on strength of glass sand concrete were reported previously by Du and Tan [63]. 

While analyzing the strength properties, slightly smaller values were observed for the concretes with LCD admixture, in relation to the reference concretes CI.0 and CII.0. The differences in the obtained values are differentiated, exhibiting no clear tendency. They are most pronounced in the tensile strength test; however, due to the high coefficient of variation (over 10%), they cannot be subjected to a comparative analysis. Generally, it can be stated that lower values were usually found in the concretes with LCD addition. Such observation can be explained both by a higher air content in the concrete mixtures with LCD admixture (the considered concretes exhibited diversified consistency with low degree of liquidity within two classes), as well as the conchoidal fracture of LCD grains. The conchoidal fracture, due to its glassy and smooth texture, deteriorates the mechanical adhesiveness of cement paste to aggregate granules, despite its low porosity found while examining the interfacial transition zone (ITZ). Nevertheless, the low share of the admixture should reduce its significance. It should also be emphasized that extending the maturation time substantially mitigates the differences in compressive strength values. Following 90 days of maturation, all concretes reached the same class. which is especially prominent while applying the CEM II/B-S 42.5 N cement that is characterized by a slower improvement of the strength parameters in relation to CEM I 42.5 R.

**Figure 6:**
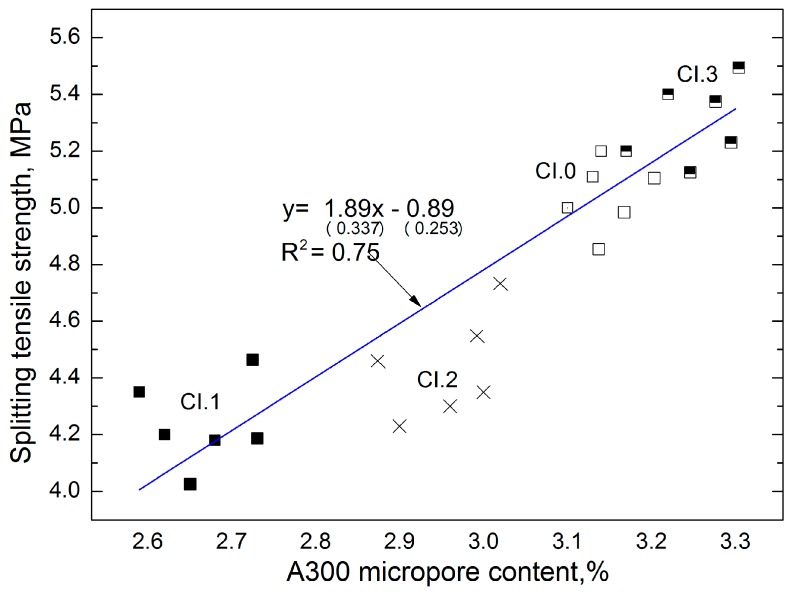
Dependence of splitting tensile strength of CI concrete on A300 micropore content.

The graphs (Figure 6) show the dependence of the different properties of CI concrete. The dependence of A300 micropore content on the splitting tensile strength for concrete is shown in Figure 6. In this study, the determined micropore content directly corresponds to the splitting tensile strength of concrete CI. The linear trend was characterized by a coefficient of correlation *R*^2^ = 0.75 and relatively low errors in the intercept. The error is considered to be small if the quotation estimator standard error and the estimator is less than 30%. In the first case it is 17% and in the second it is 28%.

**Figure 7:**
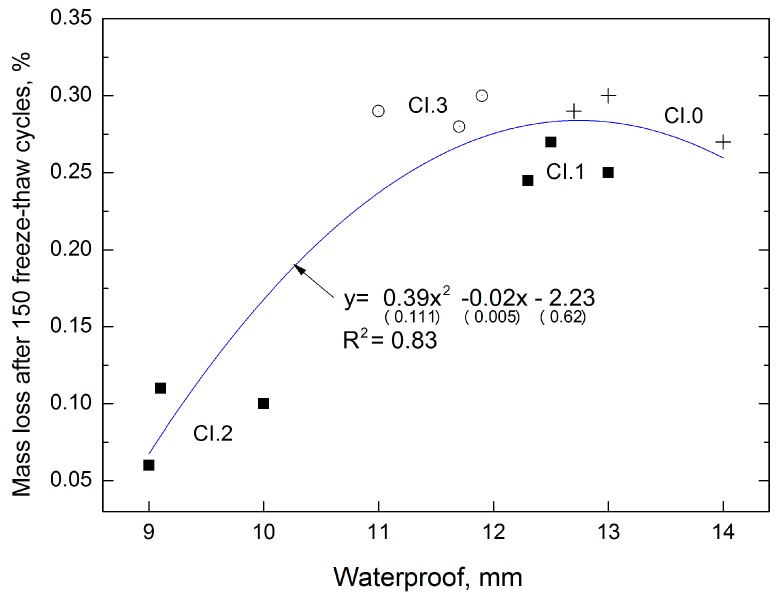
Dependence between water-tightness and mass loss after 150 freeze–thaw cycles of CI concrete.

The following model (Figure 7) presents the extent to which the characteristic of CI concrete affects the frost resistance the indirectly defines the corrosion resistance of the material. Figure 7 shows the model as well as frost resistance (mass loss of samples after 150 freeze–thaw cycles) features of concrete depending on the water resistance (water penetration).

The obtained correlations can be described by the equation $y=0.39x^{2}-0.02x-2.23\;$, which is characterized by a coefficient of determination *R*^2^ = 0.83 and relatively low errors in the intercept (respectively 28%, 25%, 28%). The higher the water-tightness, the higher the frost resistance and the lower the mass loss. The reference concrete CI.0 is characterized by the lowest water-tightness (deepest water penetration), which corresponds to the highest mass loss during the water tightness test. An increase in LCD admixture to 2% resulted in 3-fold decrease in the mass loss of the samples.

**Figure 8:**
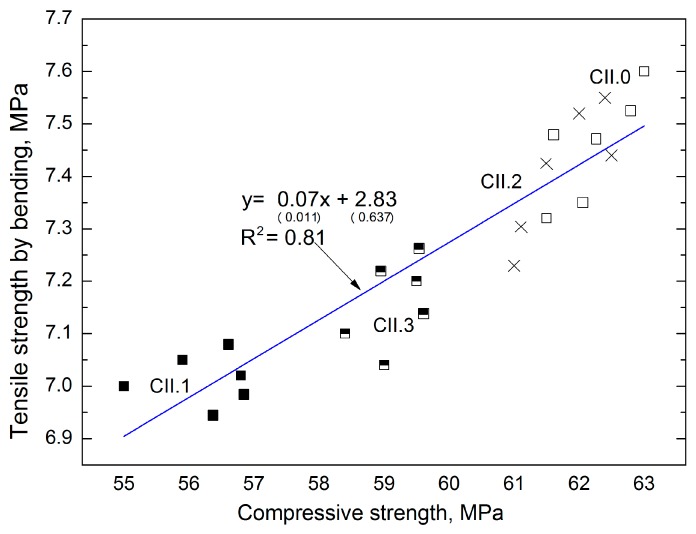
Dependence between the compressive strength and tensile strength in bending of CII concrete.

The above-mentioned dependences were not observed in the concretes with CII cement. In turn, good correlation was obtained between the compressive strength and tensile strength in bending (Figure 8). The linear function allows a good coefficient of determination, equal to 0.81 and low errors in the intercept (respectively 14.5%, 22.5%).

Based on the research of Batayneh et al. [64] concerning the impact of various waste materials on the properties of concretes and hardened concrete, it was found that the use of aggregate made of recycled concrete, due to the shape and texture of the grain surface, significantly reduces the workability of the concretes. Similar observations were made when using crushed plastics. In our own research, the impact of LCD additive with significantly smaller amounts and completely different mineralogical and chemical characteristics did not significantly affect the consistency of the mixtures.

In turn, the use of crushed glass as a fine aggregate increases the compressive strength of concrete, but due to the long-term increased impact of alkali on cement paste, the durability of concrete should be taken into account. This is a very important aspect of the concrete durability, however, due to extensive chemical analyzes carried out in relation to the tested concretes, according to the authors, it is irrelevant [64].

The use of 20% recycled plastics or concrete aggregate according to Batayneh et al. [64] reduces the compressive strength compared to reference concrete with natural aggregate. Despite the small amounts of LCD addition used in our studies, similar relationships in compressive strength values were observed in the early maturing periods (see Figure 5). However, after a longer maturing period (90 days), the same compressive strength classes of the tested concretes were obtained.

As a result of research of Mahesh and co-workers [65] regarding the impact of waste from polyethylene plastics, it was found that despite the reduction of early compressive strength of tested concretes (5–10% of the used waste), their compressive strengths after 28 days were comparable to the reference concrete [65]. Similar correlations were observed in own research after the use of LCD as an additive (Table 10).

In water permeability studies of concretes substituting a portion of fine aggregate (4–12%) with glass waste, an increase in the water permeability of concrete was observed along with the increase of the additive added [66]. Similarly, in the present research this increase also occurred, but did not exceed 0.3%.

### 4.4. Scanning Electron Microscopy with Energy Dispersive Spectroscopy

The micro-structure study of an LCD panel (Figure 9 and Figure 10) as well as CI.0, CI.1, CII.0, and CII.1 (Figure 11, Figure 12, Figure 13, Figure 14, Figure 15, Figure 16, Figure 17 and Figure 18) combined with a local analysis of chemical composition (EDS) aimed at determining the morphology and texture of created forms and identification of basic chemical components.

**Figure 9:**
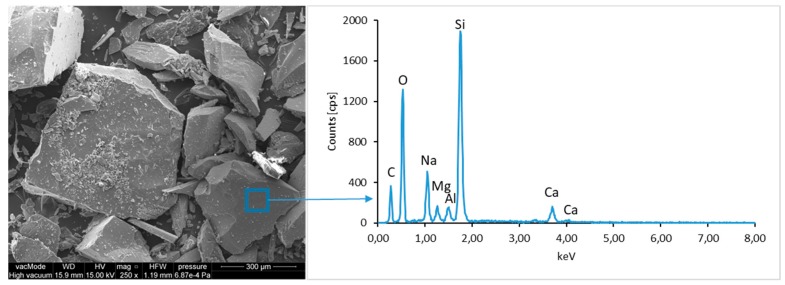
Scanning electron microscopy (SEM) micro-structure of an LCD panel and results of elemental analysis in energy dispersive spectroscopy (EDS) micro-area.

**Figure 10:**
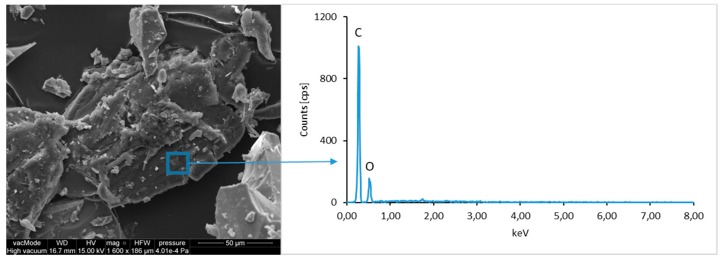
SEM micro-structure of an LCD panel and results of elemental analysis in EDS micro-area.

The ground LCD panels are characterized by a sharp-edged, irregular shape showing characteristic conchoidal fracture surfaces typical for crystalline phases. The panel elements indicate highly diversified grain size distribution, including grains up to 2 mm, 300–500 µm, and 20–100 µm. The chemical composition includes silicon, sodium and calcium oxides, the presence of which is connected with the glassy surface of an LCD panel (Figure 9). Additionally, organic carbon bonds are found in the waste material, originating from the plastic elements of LCD panels (Figure 10).

**Figure 11:**
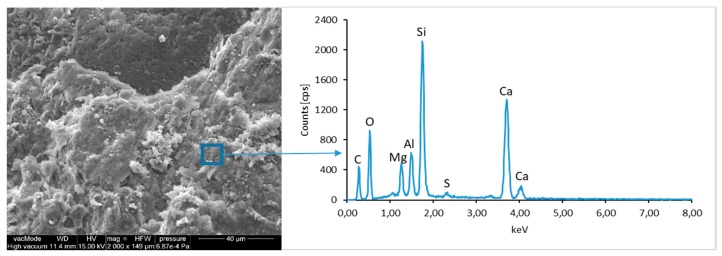
SEM micro-structure of CII.0 concrete and results of elemental analysis in EDS micro-area—compact clusters of C–S–H phase particles can be seen.

**Figure 12:**
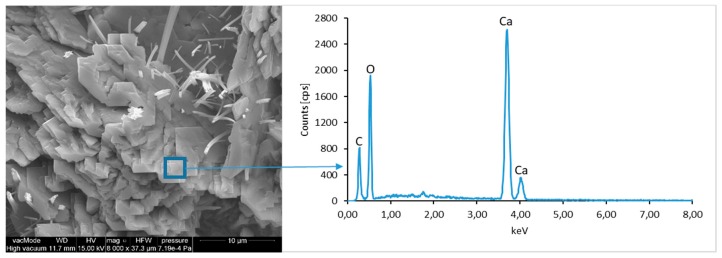
SEM micro-structure of CII.0 concrete and results of elemental analysis in EDS micro-area (portlandite)—well-formed portlandite crystals with C–S–H phase fibers are visible.

**Figure 13:**
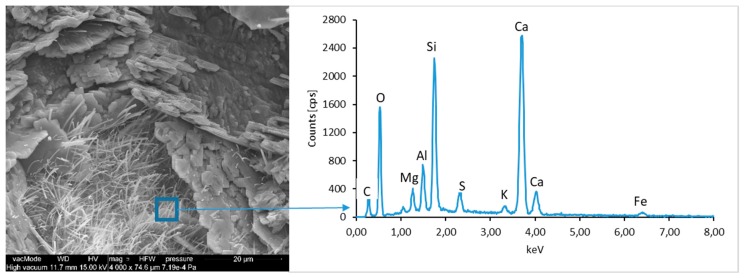
SEM micro-structure of CII.0 concrete and results of elemental analysis in EDS micro-area—well-developed portlandite and C–S–H phase crystals are shown.

The morphological analysis of pastes and their elemental compositions indicates that the main components of concretes involve calcium, aluminum and silicon oxides. The C–S–H phase, found in the cement paste hydration products, is an important component due to its dominant share. The observation of fractures of considered concretes using a scanning electron microscope with EDS analysis show different forms of C–S–H phase. The conducted studies indicate that in CII.0 concrete, the hydration products mainly include irregular and isometric or flattened particles forming relatively compact aggregations that correspond to III and IV morphological types of C–S–H phases, according to the Diamond’s classification (Figure 11). In the remaining concretes, i.e., CII.1, CI.0 and CI.1, the morphology of C–S–H phase samples indicates type I in the Diamond’s classification that corresponds to well-crystallized fibrous structure (Figure 12, Figure 13 and Figure 18). The needle-like forms taper at their ends and intertwine, filling the space between them. The length of C–S–H fibers amounts to 0.5–10 µm and their diameter amounts to about 2 µm.

**Figure 14:**
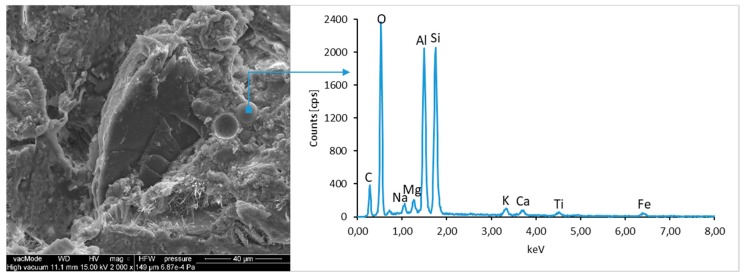
SEM micro-structure of CII.1 concrete and results of elemental analysis in EDS micro-area for spherical forms of fly ash, angular LCD forms and needle-like forms of C–S–H phase are visible.

**Figure 15:**
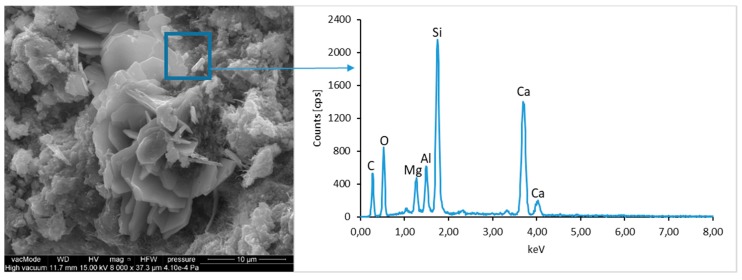
SEM micro-structure of CII.1 concrete and results of elemental analysis in EDS micro-area for visible lamellar portlandite forms forming a rosette embedded in C–S–H phase.

**Figure 16:**
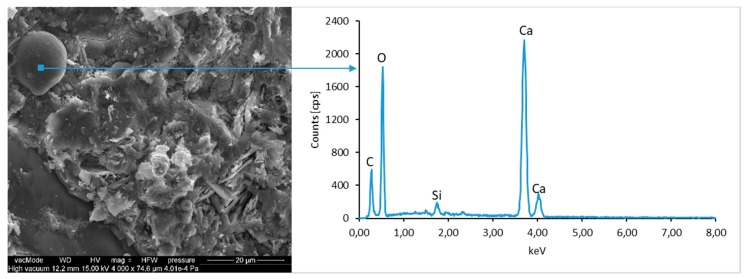
SEM micro-structure of CI.0 concrete and results of elemental analysis in EDS micro-area for visible portlandite grains, a fly ash particle and C–S–H phase can be seen.

**Figure 17:**
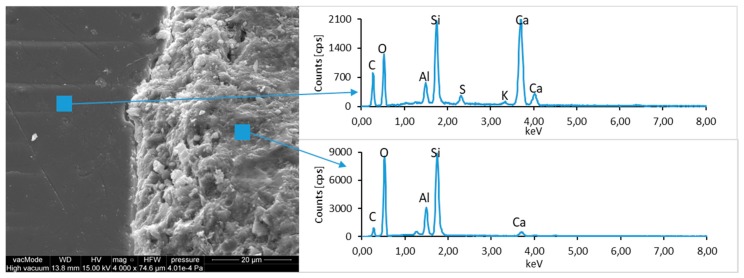
SEM micro-structure of CI.1 concrete and results of elemental analysis in EDS micro-area (1—glassy LCD; 2—paste; strong interface between them is apparent.

**Figure 18:**
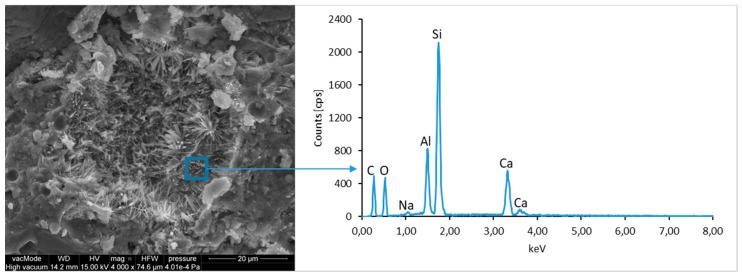
SEM micro-structure of CI.0 concrete and results of elemental analysis in EDS micro-area—fibrous structure of C–S–H is visible in the center.

The calcium hydroxide Ca(OH)_2_ (portlandite) found in cement pastes forms hexagonal lamella with the thickness of about 0.5 µm. Lamellar, rosette-forming aggregations of portlandite are embedded into the C–S–H phase (Figure 13 and Figure 15). The micro-stricture of the paste corresponding to the concretes with fly ash addition is more compact, which results from the increased content of C–S–H phase being a product of a reaction between aluminosilicate enamel and calcium hydroxide from hydrolysis of silicate phases. It is characterized by the presence of characteristic spherical fly ash forms, which are well-preserved particles with the diameter of approximately 20 µm (Figure 14 and Figure 16). The observation of paste–LCD panel interface showed that the area in its vicinity is tightly filled (Figure 17). This layer does not exhibit increased porosity and no scratches or cracks are visible; thus, the paste is characterized by good adhesion to the LCD panel.

### 4.5. Leaching Test

The analysis of the obtained results pertaining to heavy metal leaching from integral/monolithic forms of concretes with LCD waste (3%) is presented in Table 11. The obtained leaching test results were compared with the permissible leaching values that constituted a criterion for the Regulation of Minister of Environment [67,68] and landfilling waste other than inert and hazardous [69].

Conducting the leaching test on all concrete samples created a strict environmental regime enabling their release and indicating a very low mobility. All considered elements in the eluates obtained using the EN 12457-4 [49] and ML methods indicated the concentration which was below the limit of detection. The chemical investigations showed that the total content of particular elements in the analyzed concrete, i.e., arsenic, zinc, strontium, barium, phosphorus, potassium, iron, titanium, and copper, did not exceed the permissible concentration for discharging wastewater to water or soil (including Group A soil, i.e., protected areas); the limit values pertaining to hazardous substances were not exceeded either [67,68]. The trace amounts of arsenic, zinc, strontium, barium, phosphorus, potassium, iron, titanium, and copper are 10^4^–10^6^ fold lower than the permissible values.

The concentrations of the elements mentioned above in the CI.3 and CII.3 concretes are at a comparable and similar level. The effect of LCD addition on concrete on leaching is practically imperceptible. Only elements and substances such as Zn, As, CH_2_, Sn, Ba, Cu were observed at the detection limit level. It was expected considering XRF results of LCD panel (Table 7) in which low concentrations of elements such as Sr, Sn, Zn, As, Ti, Ba, Sb were noted. In addition, adding LCD up to a maximum of 3% to the weight of concrete can only slightly affect the chemical composition of concrete without lowering its class.

Concrete matrix that immobilizes trace amounts of the elements found in LCD prevents their release into the environment and mitigates the risk of hazardous substances infiltrating into soil. This is confirmed by the test of water permeability through concrete, in which no water seepage through the samples occurred under the maximum set pressure.

The physicochemical properties of concrete, connected with the specific properties of cement hydration products (main component of concrete) and the concrete production technology make it one of the best environments for an efficient management of industrial wastes. Hydrated calcium silicates, especially C–S–H phase, are responsible for the immobilizing properties of cement pastes (including the resulting mortars and concretes) [70,71,72,73]. The wastes managed in this way are much more geochemically stable than traditionally landfilled wastes. This solution contributes to reducing the area required for waste storage, which is directly connected with decreasing the environmental fees incurred by the enterprises landfilling LCD wastes.

## 5. Conclusions

The physical properties and micro-structure of concrete with LCD waste were investigated. Studies were performed to determine the impact of LCD waste on the freeze–thaw resistance and the water-tightness of concrete. On the basis of the results and discussions presented in this paper, the following conclusions can be drawn:

- All concretes achieved the same class, i.e., C50/60 after 90 days of maturation, regardless of the LCD admixture, even though they previously belonged to different classes.
- All concretes exhibited good freeze–thaw resistance after 150 F–T cycles. The mass loss of concrete samples did not exceed 0.3% (the limit value amounts to 5%).
- The strength of concretes subjected to 150 F–T cycles dropped by 3.8% at most in the case of CI, and by 0.7% for CII with CEM II/B-S 42.5 N cement (limit value of 20%).
- The infiltration depth for all considered concretes is lower than 1.5 cm, which is a very good result. The better the water-tightness, the higher the frost resistance and the lower the mass loss.
- The ITZ between the LCD aggregate and the cement matrix is compact and free of micro-scratches or cracks.
- On the basis of the conducted studies and analysis it was proven that the waste obtained from liquid crystal display panels can be used as fine aggregate.
- The leaching tests of concrete samples indicated that the concentrations of selected elements do not exceed the limit values and their content is minimal. Therefore, taking into account the environmental standards, it can be stated that the concrete with LCD admixture is an ecological building material of full value.

## Figures and Tables

**Table 1:** CEM I 42.5 R cement clinker with its chemical composition and alkali content (%).

Compound	CaO	SiO_2_	Al_2_O_3_	Fe_2_O_3_	MgO	Na_2_O	K_2_O	Na_2_O_eq_
Content	64.41	20.23	3.62	4.36	1.36	0.26	0.55	0.63

**Table 2:** Chemical composition and alkali content in CEM II/B-S 42.5 N cement clinker (%).

Compound	CaO	SiO_2_	Al_2_O_3_	Fe_2_O_3_	MgO	Na_2_O	K_2_O	Na_2_O
Content	53.22	28.04	6.54	2.31	4.39	0.31	0.59	0.70

**Table 3:** CEM I 42.5 R cement and its physical properties.

Soundness (Le Chatelier) mm	Specific Surface cm^2^/g	Specific Gravity, kg/dm^3^	Initial Setting Time, min	Heat of Hydration J/g	2-day Compressive Strength, MPa	28-day Compressive Strength, MPa
0.8	4049	3.07	190	306	31.0	60.5

**Table 4:** Physical properties of CEM II/B-S 42.5 N cement.

Soundness (Le Chatelier) mm	Specific Surface cm^2^/g	Specific Gravity, kg/dm^3^	Initial Setting Time, min.	Heat of Hydration J/g *	2-day Compressive Strength, MPa	28-day Compressive Strength MPa
1.0	4618	2.95	260	270	21.1	57.9

* Heat of hydration measured after 41 h with the use of semiadiabatic method.

**Table 5:** Sieve analysis of the aggregate as well as liquid crystal display (LCD) waste, %.

Sieve Size, mm	Sand	Gravel	LCD
8	100	100	100
4	100	45.3	100
2	96.4	14.3	72.1
1	82.8	2.1	33.5
0.5	52.2	0.9	10.9
0.25	10.1	0.5	6.4
0.125	0.7	0.2	1.5

**Table 6:** Composition of concrete mixtures.

Concrete Components	Concrete							
	CI.0	CI.1	CI.2	CI.3	CII.0	CII.1	CII.2	CII.3
Cement CEM I 42.5 R, kg/m^3^	358	358	358	358	-	-	-	-
Cement CEM II/B-S 42.5 N, kg/m^3^	-	-	-	-	374	374	374	374
Fly ash, kg/m^3^	53	53	53	53	-	-	-	-
Sand 0/2 mm, kg/m^3^	897	893	890	887	916	912	908	904
Gravel 2/8 mm, kg/m^3^	1033	1033	1033	1033	1055	1055	1055	1055
LCD admixture, kg/m^3^	-	3.58	7.16	10.74	-	3.74	7.48	11.22
Superplasticizer, L/m^3^	2.877	2.877	3.22	3.32	1.870	1.870	2.99	3.09
Plasticizer, L/m^3^	2.261	2.261	2.261	2.261	1.870	1.870	1.870	1.870
Aerating admixture, L/m^3^	0.045	0.045	0.045	0.045	0.041	0.041	0.041	0.041
Hydrophobizing admixture, L/m^3^	2.055	2.055	2.055	2.055	2.055	2.055	2.055	2.055
Water, L/m^3^	122	122	122	122	127	127	127	127
*w*/*c*	0.34	0.34	0.34	0.34	0.34	0.34	0.34	0.34
*w*/*b*	0.30	0.30	0.30	0.30	0.34	0.34	0.34	0.34

**Table 7:** Chemical composition (%) of an LCD panel.

**Component**	**SiO_2_**	**Al_2_O_3_**	**CaO**	**MgO**	**P_2_O_5_**	**Cl**	**K_2_O**	**Fe_2_O_3_**	**Zn**	**As**
Concentration, %	24.64	5.10	2.68	0.54	0.08	0.04	0.03	0.05	0.03	0.17
**Component**	**Ba**	**Sr**	**Zr**	**Ag**	**Sn**	**Sb**	**Ti**	**Cu**	**I**	**CH_2_**
Concentration, %	0.21	1.00	0.26	0.05	0.02	0.03	0.01	0.01	0.01	65.12

**Table 8:** Results obtained from concrete mixtures.

Property	Mixture							
	CI.0	CI.1	CI.2	CI.3	CII.0	CII.1	CII.2	CII.3
consistency	V0/V1							
air content, %	5.6	6.0	6.0	5.9	5.7	6.2	6.0	5.9
density, kg/m^3^	2325	2315	2310	2318	2300	2309	2301	2305

**Table 9:** Mean values of open porosity, volumetric density and water absorption of concrete.

Physical Properties	Concrete							
	CI.0	CI.1	CI.2	CI.3	CII.0	CII.1	CII.2	CII.3
Volumetric density ρ_V_, kg/m^3^	2294	2276	2274	2210	2297	2280	2287	2209
Concrete porosity according to EN 480-11 [54]	-	-		-	-	-	-	-
Total air content A, %	6.12	5.54	6.21	5.80	5.31	5.44	5.09	5.25
Specific surface of air pores system α, mm^−1^	25.31	23.21	21.98	24.32	20.05	20.95	20.55	20.88
Spacing factor L, mm	0.18	0.16	0.17	0.13	0.12	0.13	0.135	0.14
A300 micropore content, %	3.14	2.96	2.59	3.22	2.18	2.32	2.41	2.38
Water absorption by weight *n*_w_, %	4.8	4.9	4.9	5.0	4.5	4.6	4.7	4.8
Water tightness according to PN-B-06250 [43]	-	-	Water tightness class W8				-	-
Water penetration, mm	13.5	12.5	9.8	11.5	17.0	15.5	13.4	13.8
Frost resistance according to PN-B-06250 [43]	-	-	Frost resistance class F150				-	-
Mass loss after 25 cycles, %	0.2	0.2	0.1	0.2	0.3	0.3	0.2	0.2
Mass loss after 50 cycles, %	0.2	0.2	0.1	0.3	0.3	0.3	0.1	0.2
Mass loss after 75 cycles, %	0.1	0.1	0.1	0.3	0.3	0.3	0.1	0.3
Mass loss after 150 cycles, %	0.3	0.2	0.1	0.3	0.3	0.3	0.2	0.3
Durability reduction after 150 cycles, %	3.7	0.1	2.2	3.8	0.3	0.7	0.6	0.7

**Table 10:** Mechanical properties of concretes.

Physical Properties		Concrete Type							
		CI.0	CI.1	CI.2	CI.3	CII.0	CII.1	CII.2	CII.3
Compressive strength after 7 days, MPa	*f*_c,cube#100_	66.8	63.2	63.5	62.7	44.0	39.8	42.6	42.4
	*f*_c,cube#150_	60.1	56.9	57.2	56.4	39.6	35.8	38.3	38.2
Compressive strength after 28 days, MPa	*f*_c,cube#100_	76.1	72.2	72.6	70.9	69.3	62.1	68.9	65.6
	*f*_c,cube#150_	68.5	65.0	65.3	63.8	62.4	55.9	62.0	59.0
**Class**		**C50/60**	**C50/60**	**C50/60**	**C45/55**	**C45/55**	**C40/50**	**C45/55**	**C45/55**
Compressive strength after 90 days, MPa	*f*_c,cube#100_	78.6	76.8	76.6	75.9	74.8	75.4	75.7	73.9
	*f*_c,cube#150_	70.7	69.1	68.9	68.3	67.3	67.9	68.1	66.5
**Class after 90 days**		**C50/60**	**C50/60**	**C50/60**	**C50/60**	**C50/60**	**C50/60**	**C50/60**	**C50/60**
Tensile strength in bending, MPa		8.32	7.85	7.76	7.64	7.55	7.05	7.52	7.04
Splitting tensile strength, MPa		5.65	4.15	4.35	5.10	4.61	4.54	4.57	4.42

**Table 11:** Comparison of the values of elements leached from an concrete without and with 3% LCD and permissible values according to selected regulations (mg/L).

Component	Concentration (mg/L)				Permissible Value *	Permissible Value **	Permissible Value ***
	CI.0	CI.3	CII.0	CII.3			
Al	0.0005	0.0005	0.0005	0.0005	<3.0	-	-
P	0.000006	0.000007	0.000006	0.000007	<1	-	-
Cl	0.000003	0.000003	0.000003	0.000003	<0.4	-	15,000
K	0.000002	0.000002	0.000002	0.000002	<80	-	-
Fe	0.000004	0.000005	0.000005	0.000005	<10.0	-	-
Zn	-	0.000002	-	0.000002	<2.0	-	50
As	-	0.000016	-	0.000018	<0.1	<20	-
Ag	0.000001	0.000005	0.000001	0.000005	<0.1	-	-
Sn	-	0.0000018	-	0.0000017	<1	<20	-
Ba	-	0.00002	0.00001	0.00002	<2	<200	100
CH_2_	-	0.00065	-	0.0007	-	<0.1	-
Ti	0.0000002	0.0000004	0.0000002	0.0000005	<1.0	-	-
Cu	-	0.0000004	0.00001	0.0000003	<0.5	<30	<50

* Regulation of Minister of Environment of 24 July 2006 on conditions to be met for the introduction of sewage into water or soil and on substances particularly harmful to the aquatic environment (*Journal of Laws*, 2006, no. 137, item 984) [67]. ** Regulation of Minister of Environment of 9th September 2002 on soil and land ground standards (*Journal of Laws*, 2002, no. 165, item 1359) [68]. *** Regulation of Minister of Economy on the acceptance of waste for landfill; base test: liquid/solid phase = 10 L⋅kg of dry matter^–1^ [69].
